# What Have We Learned From the Typhoid Fever Surveillance in Africa Program?

**DOI:** 10.1093/cid/civ675

**Published:** 2016-03-01

**Authors:** Stephen Baker, Joachim Hombach, Florian Marks

**Affiliations:** 1 Hospital for Tropical Diseases, Wellcome Trust Major Overseas Programme, Oxford University Clinical Research Unit, Ho Chi Minh City, Vietnam; 2 Centre for Tropical Medicine, Oxford University; 3 London School of Hygiene and Tropical Medicine, United Kingdom; 4 Initiative for Vaccine Research, World Health Organization, Geneva, Switzerland;; 5 Department of Epidemiology, International Vaccine Institute, Seoul, Republic of Korea

**Keywords:** *Salmonella* Typhi, sub-Saharan Africa, epidemiology

## Abstract

The Typhoid Fever Surveillance in Africa Program (TSAP) was established in 2009 to fill the data void concerning invasive *Salmonella* disease in sub-Saharan Africa, and to specifically estimate the burden of bloodstream infections caused by the key pathogen, *Salmonella enterica* serovar Typhi. TSAP has achieved this ambitious target, finding high incidences of typhoid fever in both rural and urban populations in several countries in sub-Saharan Africa. The results of TSAP will undoubtedly dictate the direction of future typhoid fever research in Africa, and at last provides a key piece of the disease burden jigsaw puzzle. With the dawn of new Vi conjugate vaccines against *Salmonella* Typhi, the next priority for the typhoid community must be providing the required data on these vaccines so they can be licensed and provided to those in high-risk groups and locations across sub-Saharan Africa.

The paucity of epidemiologic data regarding invasive *Salmonella* disease in sub-Saharan Africa led the World Health Organization (WHO) in 2008 to call for a continent-wide approach in generating more accurate disease incidence and antimicrobial susceptibility data. In 2009, the Bill & Melinda Gates Foundation provided the International Vaccine Institute funding to launch the Typhoid Fever Surveillance in Africa Program (TSAP). The aim of TSAP was to fill the data void regarding invasive *Salmonella* disease in sub-Saharan Africa and specifically to estimate the burden of bloodstream infections caused by the key typhoidal pathogen, *Salmonella enterica* serovar Typhi. TSAP was established to provide these data from 13 sentinel sites in 10 countries in sub-Saharan Africa. It was predicted that TSAP would provide contemporary information to assess whether disease incidence was sufficient to warrant the introduction of preventive measures, such as vaccination.

Prior global estimates for the burden of invasive *Salmonella* disease found that South-Central and East-Central Asia experienced the highest incidences of typhoid fever, with >100 cases per 100 000 person-years of observation [1]; Africa was previously estimated to have a moderate incidence (10–100 cases/100 000 person-years of observation) [2]. The primary data from TSAP expand these previous estimations (F. Marks, V. von Kalckreuth, P. Aaby, et al, submitted), and describe remarkably high incidence rates in 3 of 13 of the African surveillance sites. Additionally, high incidence rates were observed specifically in children <15 years of age in 4 of 13 sites, and in at least 1 age group in 6 of 13 sites (F. Marks, V. von Kalckreuth, P. Aaby, et al, submitted). These figures are comparable to the earlier Diseases of the Most Impoverished program conducted in Asia [1], and again show that children between the ages of 2 and 14 years bear the global brunt of the burden of typhoid fever. Significantly, the incidence rates of typhoid fever in many of the TSAP sites were equivalent to, or indeed greater than, incidences reported in parts of Asia in the early 2000s [1, 3].

The findings and study methodology described in this supplement of *Clinical Infectious Diseases* will undoubtedly influence the direction of future typhoid fever research in Africa and outline many key observations regarding typhoid fever epidemiology on this continent. In addition to high incidence rates, the focal (site-specific) nature of the disease in Africa will have substantial implications on the impact of potential intervention strategies. In the supplement article by Cruz and coauthors, it is shown that the incidence of typhoid fever in Ghana was substantially different between adjacent urban and rural areas [4]. Indeed, a recurring TSAP theme is the variable incidence of typhoid fever between sites, with many rural populations having similar or higher incidences than urban locations. These data show that structural improvements in water and sanitation infrastructure, as well as possible vaccination campaigns, should not be limited only to densely populated settings.

An additional key TSAP finding was the substantial prevalence of drug resistance and multidrug-resistant strains of invasive *Salmonella* spp. circulating in sub-Saharan Africa (F. Marks, V. von Kalckreuth, P. Aaby, et al, submitted) [5]. Notably, a high prevalence of resistance to first-line antimicrobial agents was identified in both *Salmonella* Typhi and nontyphoidal *Salmonella* isolates in some locations. These data are largely reflective of other observations in further sites in Africa and Asia [6–9]. However, the scale of multidrug-resistant strains is concerning and likely to be exacerbated by the combination of the burden of non-specific febrile disease with a lack of diagnostic resources, which leads to the imprecise usage of antimicrobials. Additional *Salmonella* Typhi genomics work, also published in 2015, has shown the spread of the H58 lineage into Africa from Asia, predicting that antimicrobial resistance is a problem that is likely to increase over time [10].

The work presented in this supplement should raise the awareness of typhoid fever in rural locations in Africa, and follow-up studies should be conducted in other rural settings that were not sampled through TSAP. However, further studies, such as this and other typhoid fever studies, are confounded by the available diagnostic testing methods [11]. Current blood culture methods for diagnosing typhoid fever require a reasonable laboratory infrastructure, which is not available in many regions of sub-Saharan Africa. The upshot in the limitations of diagnosis is an inability to assess the burden of invasive *Salmonella* disease in remote settings with poor water quality and sanitation, where high disease burdens may now be anticipated. An inability to detect patients in remote settings results not only in an underestimation of the burden of invasive *Salmonella* disease, but also the associated disease severity and mortality. Ongoing investments by the Bill & Melinda Gates Foundation and other donors will hopefully yield better diagnostic tests that can be used at the point of care without the need for cumbersome and insensitive blood culture methods [12].

Invasive *Salmonella* disease is most prevalent in underprivileged communities with poor quality water supplies and limited sanitation. Often these communities are overcrowded and have restricted access to medical care. Preventive vaccination holds particular potential in reaching marginalized populations. However, in 2011, the Gavi Alliance stated that no financial support would be provided to countries supporting the introduction of the presently available Vi polysaccharide vaccines due to insufficient data on disease burden and limited vaccine efficacy in children aged <2 years. The lack of Gavi subsidy, combined with suboptimal performance of Vi polysaccharide vaccines, led to only very limited use of these vaccines. Five years later, 2 typhoid Vi conjugate vaccines indicated for use in children aged ≥2 years have been licensed in India, and several more are in development [13]. Two preconditions have to be met for Gavi to consider subsidy of typhoid conjugate vaccines: (1) WHO prequalification of a typhoid conjugate vaccine and (2) WHO recommendations for use in form of a position paper. For WHO to act, relevant data on the clinical performance of the new vaccines need to be published, and manufacturers need to show interest in international use of the vaccine and submit their data file for prequalification. The TSAP study results show a great need for these vaccines, and hopefully provide further impetus to move the agenda to make these vaccines available to the populations in greatest need.

Possible future vaccination strategies in Africa include preemptive, responsive (in the case of an outbreak), and food handler immunization [14]. However, to motivate policymakers to actually deploy vaccines against invasive *Salmonella* disease, the research community must communicate not only the disease burden but also the effectiveness of vaccination, and the country-specific risk factors necessary to identify target groups/areas. Current research efforts are aimed at developing models that predict risk factors across sub-Saharan Africa to facilitate the assessment of possible *Salmonella* disease burden. However, such models can never replace real disease burden data in at-risk countries and communities, and it is unlikely that health ministries will approve mass vaccination programs on the basis of modeling data alone. Therefore, we again highlight the need for functional diagnostic tests and sustained disease surveillance alongside the genomic tracking of organisms and genes facilitating antimicrobial resistance.

Perhaps the most critical next activity in invasive *Salmonella* disease research is the identification of sites in Africa and Asia with consistently high burdens of disease for which water, sanitation, and hygiene (WASH) improvements can be implemented in conjunction with, or in comparison to, mass vaccination. Such studies would begin to quantify a more precise measure of the impact of WASH on disease incidence, and determine the extent of vaccination coverage alongside WASH improvements required for location-specific elimination of invasive *Salmonella* disease. Such data on the impact of the interventions will provide the most compelling case for decision makers to act upon.

In conclusion, data from the TSAP program have firmly positioned invasive *Salmonella* disease as a public health priority on the African agenda. With the dawn of Vi conjugate vaccines and now-reliable incidence figures in Africa, the typhoid fever community really now needs to tackle the political issues surrounding typhoid fever immunization if we truly believe that regional elimination is a tangible target.
